# Adaptive robotic system for the inspection of aerospace slat actuator mount

**DOI:** 10.3389/frobt.2024.1423319

**Published:** 2024-06-27

**Authors:** Nour M. Morsi, Mario Mata, Colin S. Harrison, David Semple

**Affiliations:** ^1^ School of Computing and Built Environment, Mechanical Engineering Department, Glasgow Caledonian University, Glasgow, United Kingdom; ^2^ School of Computing and Built Environment, Computing Department, Glasgow Caledonian University, Glasgow, United Kingdom

**Keywords:** robotic systems, inspection, aerospace, machine learning, drill analysis, slat actuator mount

## Abstract

**Introduction:** Robotics uptake in the aerospace industry is low, mainly due to the low-volume/high-accuracy production that aerospace manufacturers require. Furthermore, aerospace manufacturing and assembly sites are often unstructured environments not specifically suitable for robots to operate in.

**Methods:** This paper introduces a robotic visual inspection system using off-the-shelf components able to inspect the mounting holes for wing slat actuators without the need for fixed-coordinate programming; the part just needs to be left within reach of the robot. Our system sets one of the opposed pairs of mounting holes as a reference (the “datum”) and then compares the tilt of all other pairs of mounting holes with respect to it. Under the assumption that any deviation in the mounting hole tilt is not systematic but due to normal manufacturing tolerances, our system will either guarantee the correct alignment of all mounting holes or highlight the existence of misaligned holes.

**Results and Discussion:** Computer-vision tilt measurements are performed with an error of below 0.03° using custom optimization for the sub-pixel determination of the center and radius of the mounting holes. The error introduced by the robot’s motion from the datum to each of the remaining hole pairs is compensated by moving back to the datum and fixing the orientation again before moving to inspect the next hole pair. This error is estimated to be approximately 0.05°, taking the total tilt error estimation for any mounting hole pair to be 0.08° with respect to the datum. This is confirmed by manually measuring the tilt of the hole pairs using a clock gauge on a calibrated table (not used during normal operation).

## 1 Introduction

Aerospace assembly processes account for 45%–60% of the total manufacturing (Jiang et al., 2021). Titanium alloys, super-alloys, and composite parts were used to improve the overall strength of the aircraft (Jiang et al., 2009). However, such components are difficult to repair and can cause instability issues during drilling (Turki et al., 2014). When manufacturing rivets and screw connections, the 1.5–3 million holes per aircraft needed for assembly are typically drilled using a twist drill (Aamir et al., 2020; Giasin and Ayvar-Soberanis, 2017). Twist drilling is a major machining process in the aerospace industry (Aamir et al., 2020). The mounting holes are pneumatically drilled and inserted into the fuselage skin. This process generates shock and vibration phenomena that can lead to fatigue failure (Sun et al., 2018). On the other hand, a hand-held pneumatic drill is used to drill mounting holes in the fuselage skin. Drilling direction that deviates from the surface normal will cause perpendicularity and hole-diameter quality issues, which is unsafe for an aircraft (Shi et al., 2016). Additionally, removing material using an edge chisel to accommodate the hole deviation creates stress concentration areas (Sun et al., 2018) that are the main cause of fatigue crack formation.

Robotics is of interest for the manufacture of aerospace systems, especially aircraft structures. Robots offer cost and flexibility advantages to manufacturers in this industry. This option increases the flexibility for drilling, unlike standard drilling systems such as large gantries, which require unobstructed access to the product. The only drawback of robotic systems is that they cannot independently meet the high-precision requirements of aerospace assembly (Devlieg and Szallay, 2010), and additional techniques like added external sensors need to be combined with the robot.

**FIGURE 1:**
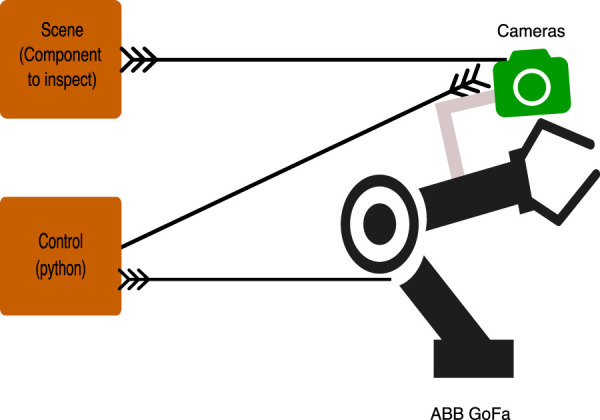
Conceptual framework of the proposed robotic system.

**FIGURE 2:**
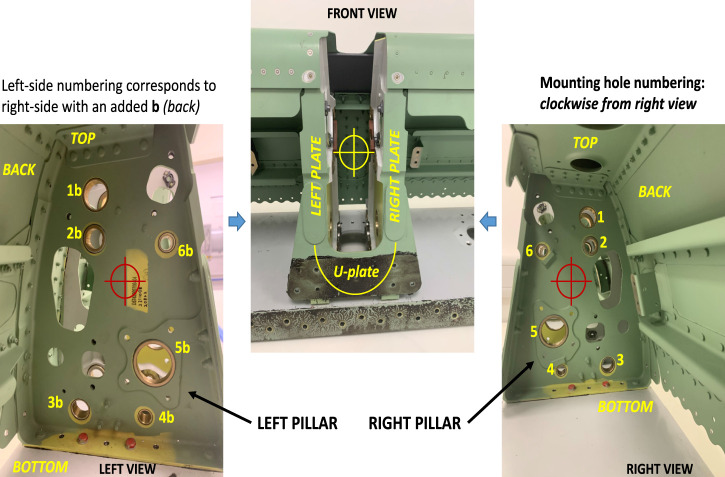
Slat support slot mounting holes, numbered.

This paper focuses on introducing a robotic system capable of determining drill hole tilts in a group of related drills in wing slat support slots, as shown in Figure 2, a process commonly carried out manually in the aerospace industry. The system introduced is controlled by a simple, finite, state-controlled, autonomous robotic system with machine vision cameras and integrated light systems. The performance of the system is evaluated by comparing its inspection results with the metrology of the drill tilt deviations. The mathematical analysis of the system was carried out and verified in previously published work (Morsi et al., 2023). However, as the system lacked positional detection of shapes, the updated system was upgraded with a machine learning system and a template matching approach to improve the system’s autonomy and accuracy. The approach proposed uses visual feedback to achieve autonomous motion using Python scripts on an ABB GoFa robot. Figure 1 shows the conceptual framework. The components introduced aids in the system’s flexibility by eliminating the need for jigs and fixtures or pre-recorded positions to inspect the workpiece; it is enough to leave the part within the field of view of the robot camera.

The remainder of the paper is structured as follows: Section 2 reviews related works on robotic inspection systems for drill holes and key components needed to achieve this; Section 3 introduces the problem setting, control approach, and simulated and experimental setup; Section 4 discusses the results; and finally, the conclusion is summarized in Section 5.

## 2 Related works

### 2.1 Industry 4.0/5.0

Industry 4.0 represents the current revolution in manufacturing. The ultimate objective is to establish smart factories (Sherwani et al., 2020; Wan et al., 2019). One of the key impacts of Industry 4.0 is the increased adoption of robotic systems, driven by the pursuit of competitiveness (Javaid et al., 2021). To further increase competitiveness, robotic systems must be capable of performing in unpredictable environments by learning from collected data through various models, i.e., autonomous robotic systems (Mukherjee et al., 2021).

### 2.2 Simulation and control

To achieve adaptability for production variations, current robotic controls need to be updated as they only fit single-purpose assemblies (Arents and Greitans, 2022). Instead, sensors can be used to collect information about the surroundings, and then automated logic and programmable planning methods are used to plan operations ahead. Such systems rely on independent judgments over possible courses of action based on information about the task and its surroundings.

To test a model and analyze its output, simulation entails creating a hypothetical or real object (Žlajpah, 2008). Robotic simulators have become an essential component of research paradigms in order to assess the algorithms’ stability, efficiency, and safety levels. In this paper, RoboDK is used. It is a universal software program that offers a simulation platform to analyze various robotic configurations (Garbev and Atanassov, 2020). The initial design of the system in a simulator minimizes the risks of collision as the setup modeled provides a glimpse of the possible interactions/collisions the system will face when deployed in the real process, which helps in prior planning for the design of the tools and feasible control approaches.

### 2.3 Aerospace industry

Aerospace industry aircraft operators receive a low return on the capital used (Büchter et al., 2022), which requires them to adapt to operational demands and unveil efficient new aircraft. Cost optimization is heavily enforced to ensure that minimal growth is maintained. This strain increased during the COVID-19 pandemic, in which all planes have been grounded. This leads some airlines to repurpose planes for cargo transport to keep at least some revenue running. However, this is mainly controlled by reduced demand and sales per weight.

In manufacturing engineering, metrology and tooling are considered separate fields. Commonly, tooling, which comprises jigs and fixtures, focuses mainly on part manipulation during assembly, with quality checks as a secondary objective (Vichare et al., 2014). Typically, for the assembly of a single aircraft type, specially designed, large steel structures are bolted to the factory floor. These structures are configured to provide precise alignment and support for the assembly process. However, the structures are very expensive and have limited ability to be used for any other product variation.

When wings are assembled, hole alignment checks need to be assessed. Practically, processed structures (that may span over 20 m) are too large to be practically inserted in and measured with a coordinate measurement machine (National Physical Laboratory, 2024). Therefore, costly, large-volume metrology (LVM) metrological equipment needs to be used on the wing.

Evidently, a shift toward flexible and light tooling from heavy, rigid structures will significantly increase assembly lines and non-recurring costs (NRCs). A viable, flexible, and fast robotic inspection system is needed to pave the way for improving the aerospace manufacturing lines. This is where the proposed system can help.

### 2.4 Non-destructive testing

Part manufacturers aim to ensure product quality during production, which ensures the product in its extended life functions as intended. Therefore, discontinuities and defects need to be avoided when producing the part. Defects in parts represent imperfections, which impede its performance, ultimately leading to failure prior to the expected life expectancy. On the other hand, similar to defects, discontinuities cannot be corrected (Gupta et al., 2022).

Non-destructive testing (NDT) offers approaches that identify and quantify the characterized failure in products to account for its current state, avoiding the intensification of the failure. The approach can be applied at all stages of the product cycle, which allows for accurate monitoring of defects if present and a better understanding of the drivers of the failure, which allows for future product optimization. Presently, the aerospace sector conducts inspections using non-destructive testing carried out by experienced technicians who examine or sample the structure to assess its quality Mineo et al., (2017). NDT is a cost-effective method for material inspection, damage characterization, and quantization (Gholizadeh and Gholizadeh, 2022). However, NDT relies on the technician’s expertise and knowledge. Successful methods include visual testing (VT), ultrasonic testing, acoustic emission, eddy current testing, infrared thermography, and laser shearography (Towsyfyan et al., 2020). VT is the oldest form of NDT. This method uses visual aids, primarily the eye, to inspect the surface. The main visual aid needed to perform VT is the human eye. There are two types of VT methods: direct VT (DVT) and remote VT (RVT). Direct VT is performed when the eye can be positioned within 25 inches of the surface of the sample being examined at a minimum angle of 30° (Allgaier and Sayler, 2011). Remote VT is performed in difficult-to-access, unlit locations. In this mode, light is supplied via a cable from an external source, and the camera sends an image or live feed to the monitor. This method offers the possibility of automating the inspection (or part of it) using computer vision techniques to either algorithmically extract relevant information or manipulate the images in a way that makes human observation easier.

Light is an important prerequisite for performing VT. The amount of light that enters the eye (or the camera lens) determines the quality of the image. Several factors, such as the surface texture, brightness, temperature, size, and shape of the object, can affect the amount of light reflected. Controlling the illumination (or even providing a particular light structure) can significantly simplify VT processes.

Human factors, such as emotional and physical effects, also influence the results of VT. Replacing human-based VT with computer vision-based VT can achieve much better performance.

### 2.5 Robotic systems

Several robotic systems have been used for inspecting and drilling holes. Typically, the repeatability of industrial robots is adequate for one direction, but repeating the same procedure (such as drilling) from multiple directions is not (Devlieg and Szallay, 2010). Radial tolerances in the order of 0.25 mm cannot be achieved due to errors such as backlash and wind-up between the feedback and the joint. Therefore, inspecting structures requires mapping the environment in which the product exists, a calculated path plan and navigation procedure, and sensing devices to collect information along the path. This emphasizes that a strong information procedure aids in an accurate inspection (Almadhoun et al., 2016).

Adaptable autonomous robotic systems offer manufacturers cost and flexibility benefits. This option offers better access to difficult drilling locations, unlike a large standard drilling system (such as a gantry) that requires a clear access route to the product. If the robotic system can be moved, it can then be used to inspect large or long-thin structures. The critical disadvantage is the inability to meet the high precision needed by aerospace assembly (Devlieg and Szallay, 2010). The aerospace industry’s stringent requirements can reach positional and normality accuracies as low as 0.1 mm and 0.5°, respectively, which is not achievable with off-the-shelf industrial robots. Therefore, solutions must include embedding sensors into the robot system that correct its position and orientation to achieve the required accuracy.

Research and application of robots in assembly tasks such as large-scale aviation components have exhibited growth. Vision-based assembly methods have been widely used in industrial applications. However, pure visual sensor combinations for high accuracy remain a challenge. A literature search was conducted to understand the types of systems introduced. The proposed systems exhibit several common approaches and alignment configurations.

#### 2.5.1 Robotics based on calibrated coordinates

The most traditional approach involves offline programming and operation with a fixed (or clamped) workpiece. This method necessitates an accurate part location in the workspace (Jayaweera and Webb, 2010). This approach minimizes alignment and positional errors by using metrological equipment to ensure the perfect alignment of the workpiece. However, it is slow to respond to the fast-paced environment of the aerospace industry, which involves frequent changes in product designs. Moreover, linked weights and applied forces can create an additional loss in the joints, which commonly creates a *panel skid* where the tool deflects by 2 mm.

Devlieg et al. (2001) introduced the ONCE robotic drilling system. It was used for drilling, countersinking, and inspecting fastener holes on F/A-18E/F Super Hornet and Boeing wing trailing edge flaps, achieving a positional accuracy of ± 1.5 mm. It is a multi-functional end-effector (MFEE) tool equipped with a re-synchronization camera to align the robot’s tool spindle with the workpiece and other process tools, achieving the required positional accuracy.

Bi and Liang (2011) utilized a fixed drilling procedure for a titanium workpiece. A holding fixture frame was used to drill the lower panel of a fuel tank model, employing ABB RobotStudio software with an IRB6640-235/2.55 achieving accuracy up to 0.2 mm.

Zhu et al. (2013) utilized offline programming to generate a path for the robot to drill holes on a wing structure, mitigating alignment errors by employing four reference hole measurements that are bi-linearly interpolated to extract the surface model of the workpiece and align the KUKA KR360 robot’s tool accurately. At a distance of 0.5 m between reference holes, the positional errors were ± 0.5 mm.

#### 2.5.2 Robotics not relying on calibrated coordinates

The systems introduced in this section focus on utilizing robotic systems integrated with sensors that control the translation or rotational position of the robot to achieve accurate drilling rather than depending on accurate, calibrated positions.

Fernando et al. (2010) used a vision module to measure and correct the hole’s linear position on a fuselage surface. Furthermore, the perpendicularity is corrected by a perpendicularity module design. The module uses a patella with a spherical joint, which creates a contact between its surface and the object, and the deviations are then measured using four linear sensors.

Gong et al. (2012) used vision positioning to locate the holes to be drilled using three laser range sensors equally equidistant on a circle around a drill point. The approach focuses on measuring a small surface on the skin to align the drill. From two tangent vectors to the surface of the skin, a normal vector is calculated using their cross-product. The system is tested on multiple curved surfaces in simulation and on a mid-fuselage covering skin, achieving an alignment accuracy of 0.02°.

Yuan et al. (2014) designed a drilling end effector equipped with four laser-ranging sensors strategically placed around the drill to measure the surface normal. By obtaining coordinates from these sensors, the method calculates the surface normal at the drilling point. Additionally, the end effector incorporates an adjusting mechanism to ensure the drill attitude meets the assembly requirements. The approach achieves ±0.5° accuracy in surface normal measurement, demonstrating the effectiveness of the adjusting mechanism in achieving the desired drilling outcomes.

In general, the offline simulation approaches reviewed above achieved high accuracy and eliminated alignment errors; previous approaches incorporated jigs and devices such as shuttle tables to accurately place process tools to be picked up by the robot, such as cameras and hole probes. As the approach requires the accurate location of the components, this design confines the system’s flexibility rather than allowing freedom in the placement and usage of the tools and limits the system’s adaptability if there are any changes in the drilling and inspection processes. Moreover, the alignment accuracy of the system degrades with time (Kiran, 2017). Most importantly, such systems cannot be used in a workspace shared with humans due to the necessary safety measures.

On the other hand, when the robot is required to adjust to the tool’s location, detection and alignment are ensured by visual and laser sensors. However, the accuracy of the alignment approaches, such as laser sensors and visual sensors, is dependent on the correct clamp of the clamping module and the accuracy of the drill detection.

In recent years, approaches have aimed at moving away from fixtures and clamps to overcome vertical alignments by using metrological devices such as laser sensors and cameras to reorient the tool with respect to the workpiece. Furthermore, the bulk of the papers introduced vast approaches that considered many possible laser configurations. On the other hand, in-depth camera approaches for detection were not discussed, only mentioned. The accuracy of the hole positioning also contributes to the quality of the hole. The use of cameras to realign the tool is not evidently explored. This can be justified by the non-trivial task of having to analyze a mostly flat and textureless surface from a 2D image but to analyze hole alignments; this could be possible as the holes themselves can act as reference points. Therefore, this paper explores the use of visual servoing as an approach to detecting drill hole tilts on an aerospace workpiece.

### 2.6 Machine learning for image processing

#### 2.6.1 Machine learning

Robotic systems can acquire experience from their interactions with their surroundings, which allows them to perform their function in a more efficient manner when exposed to the same experience (Trovato et al., 2016). The cognition should be sufficient for the system to actively execute its tasks in an unstructured environment. To improve the system, a possible approach is to incorporate machine learning (ML) to address challenging tasks.

ML is a new approach used to complete a desired task without being programmed to the literal case (i.e., hard coding) (El Naqa et al., 2015). Through a repeated learning experience with the desired results attached (training), the system is created as a “soft code” in such a way that it automatically alters its actions to fit the needs required. This is implemented as an algorithm that optimizes a parametric model in such a way that it produces the desired outputs based on input examples. During the training, the ML system is able to optimize the internal parameters to produce the desired outcome for the input; this is then checked during the testing phase and, eventually, monitoring real-world operation. The ML system must be able to generalize the model built on the training data in such a way that it learns and “understands” (models) the connection between the training data and the desired outcome with enough flexibility to successfully replicate this operation on unseen data (testing data and real-world operations).

#### 2.6.2 Locating objects in the image

To accurately localize the position of a robot in uncalibrated environments, visual odometry (VO) has been proposed. It allows us to solve this problem without any prior processing of the robot’s motion (Kostavelis et al., 2016). VO estimates the relative motion between the robot and some reference elements in the image. VO has been utilized in robotic applications such as path planning, localization and mapping, and collision avoidance.

Image matching is a visual task that can be used to calculate VO (Zhang et al., 1995). Various methods have been proposed, which mainly fall into two categories:

• Template matching: correlation of image patches from a template with the target images.
• Feature matching: extraction of features from the template (such as edge segments, contours, corners, or other salient elements) and matching them with the target images.

A template may appear with some variance due to added noise, different viewpoints, and changes in illumination (Brunelli, 2009). Template matching is quite straightforward, but it is sensitive to scale and rotation that occur under different viewpoints. If this is likely to happen, feature matching increases the robustness of detections (Bay et al., 2006). *Feature vectors* are built by appending the information from the considered features; they are obtained from one (or several) *template images* as an internal representation of the object to be found. The feature vector needs to be distinctive and resistant to noise, geometric, and photometric deformations. It can then be matched against the same set of features on new images to find whether the object is present (and where it is located in that case). The matching between feature vectors from the template and any new image is based on some distance metric (like the Euclidean or Mahalanobis distance) between the template vectors.

Several papers have already proposed interesting point detectors (Harris and Stephens, 1988; Matas et al., 2004; Tuytelaars et al., 2013; Ke and Sukthankar, 2004; Mikolajczyk and Schmid, 2002; Lindeberg, 1996). Scale-invariant feature transform (SIFT) calculates the spatial and frequency of well-localized features in the image, which are invariant to image scaling and rotation (Lowe, 2004). SIFT computes a histogram of local gradients around the points of interest and stores it as a 128-dimensional vector in bins. Its uniqueness and speed make it one of the most appealing practical descriptors. The SIFT descriptor mixes localized features with distributed gradient-related features to create distinctive criteria while avoiding false detections. However, the high dimensionality of the descriptor is a major drawback (Tuytelaars et al., 2013). Further related proposals aiming to speed up the process have been devised, although at the cost of decreasing accuracy (Fergus et al., 2003). Speeded-Up Robust Features (SURF) is another promising feature detector that is based on the Hessian matrix but with a basic approximation similar to the difference of Gaussians (Lakemond et al., 2009). This detector relies on image integrals to reduce processing time. The SURF descriptor is similar to that of SIFT in that it computes reproducible orientation information about a circular region around the interest point. The region is then mapped with an aligned square region to extract the SURF descriptors. A variant introduced with SURF, Upright SURF (U-SURF), is a scale and orientation invariant detector that can compute detections at faster rates, provided that the applications it is used in do not exhibit major orientation changes.

After extracting the features, the next step is matching them with image points on a new image to find a match. A number of matching algorithms can be used. In matching algorithms, the underlying computational expense is searching for possible matches of high-dimensional vectors.

A brute-force matcher computes and compares the Euclidean distance of all of the descriptors in the new image to the descriptors of the template image, which is obviously time-consuming.

A common approach is nearest-neighbor matching (Muja and Lowe, 2014). In a metric space *M* with a multitude of points *S* = {*s*_1_, *s*_2_, … , *s*_*n*_}, the aim is to locate the element closest neighbor to the query point *q*, where *q* ∈ *M* and *NN*(*q*, *S*) ∈ *S* according to a distance function *d* minimizing *d* (*q*, *s*). The basis of nearest neighbor approaches is to pre-compute the existing dataset *P* so that the matching operation *NN*(*q*, *P*) is carried out adeptly. By finding the closest *K* points from the point of query, k-nearest neighbor (kNN) is used (Muja and Lowe, 2014). kNN is an improvement of the brute-force matcher, and it is not restricted to matching using the Euclidean distance and allows for the user to decide the number of best matches for each key point (Garcia et al., 2010).

## 3 Materials and methods

### 3.1 Slat actuator mounting slot inspection

The aim of this paper is the inspection of the mounting holes of a wing slat actuator support to detect misalignments that could prevent mounting. The frontal and side views of this support are shown in Figure 2 and Figure 5, respectively, where the mounting holes are labeled with arbitrary reference numbers, where “b” represents the back hole (e.g., 2 and 2b and 3 and 3b). Each pair of front and back holes forms an axis that corresponds to the rod inserted during assembly. The unique shape of the structure presents a significant challenge for automation due to the location of mounting holes being obscured inside the arching structure of the wing spar and the unstructured setup in which the spar itself will be located. Inside the support slot, six mounting holes on each side forming an axis require an inspection to ensure that the holes are correctly oriented before the assembly of the slat actuators can be completed.

The robotic system is required to autonomously inspect the mounting holes regardless of their position, i.e., in an uncalibrated setup; the only restriction is that the mounting slot must be within the field of view of the approach camera. The robot can then be mounted on a cart, and a person would need to place it in the vicinity of the support. The selection of a collaborative robot enables the operator to safely work next to the robot without security cages.

To check if all the rods will fit into the slot using our approach, holes 5a and 5b are used as a datum reference axis; the robot centers and orients the optical axis of the camera until it is aligned with the front hole and the tilt angle between the front and back holes is less than the threshold (0.2°). Then, the tilt in the datum reference (if present) is compared with the rest of the hole axis, indicating the amount of tilt of each pair of mounting holes, and the tilt angle is calculated using the approach proposed by Morsi et al. (2023) (mentioned in Section 3.3.1). The cart can be easily moved along a hangar to inspect several wing spars lying, around with no particular positioning requirement other than room enough for the cart to move along them. Instead of a person pulling the cart from support to support, a simplistic, uncalibrated drag system moving the robot along the wing spar could be easily deployed; note that this fully avoids the need for a precise motion control rail.

The objectives of this paper are as follows:

• To devise a flexible motion approach to allow the accurate positioning of the tool with respect to the support.
• To integrate the machine learning approach with the motion approach to allow accurate rotation adjustment.
• To integrate the hole tilt analysis approach into this new system.

The use of a vision system coupled with a complicated shape favors approaches such as machine learning, as indicated in Section 2.6 The proposed system comprises a custom-designed, lightweight robotic arm end-effector tool holding two cameras, three light sources (controlled by a microcontroller), and a collaborative robot controlled using Python code, with an overall control approach loosely based on a finite-state machine (FSM) approach.

**FIGURE 3:**
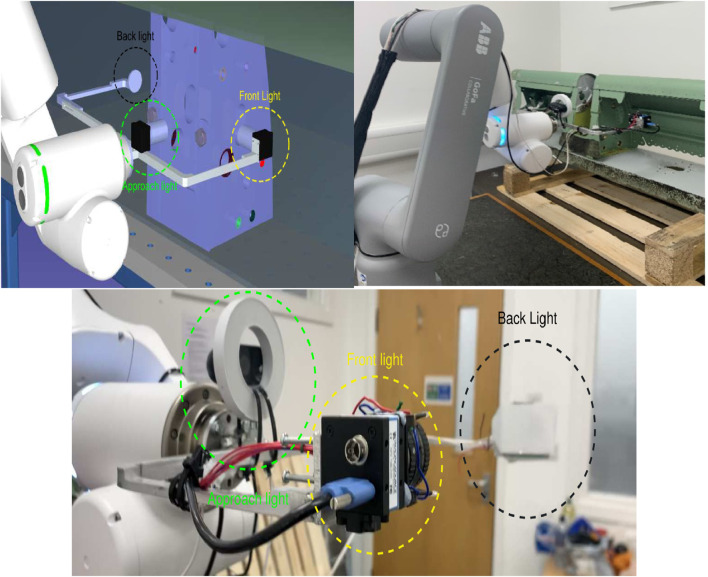
Simulated setup *versus* real setup.

To safely test the designed tool before using it on a real robot, a rough design of the slat support was modeled in SolidWorks to study the possible robot trajectories. The conceptual robotic inspection system is designed by RoboDK Inc. (2023), where simulation tests are performed to ensure that a reliable inspection system is designed before transferring actual commands to the robot. Figure 3 shows the inspection tool in RoboDK and real life, showing both its conceptual overview and its real-world implementation, along with a side view for additional detail. The three light sources and two cameras are related to each other as follows:

• Approach light: An LED ring around the approach camera, used for locating and approaching the slat support and orienting the tool with respect to it. This camera is just a basic HP webcam, mounted looking “forward,” i.e., its optical axis is roughly normal to the flange frontal surface of the robot.
• Front light: It comprises four LEDs and is mounted around the hole inspection camera, an industrial (DFK 42BUC03) camera (The Imaging Source, 2023). The intensity of the LEDs can be controlled via Pulse-width modulation (PWM) using the microcontroller. It is used for the detection and inspection of front holes (holes 1–6, on the right-hand side of the mounting slot).
• Back light (diffuse): It is intended to provide backlight for the detection and inspection of back holes (holes 1b–6b on the left-hand side of the mounting slot) using the frontal inspection camera.

**FIGURE 4:**
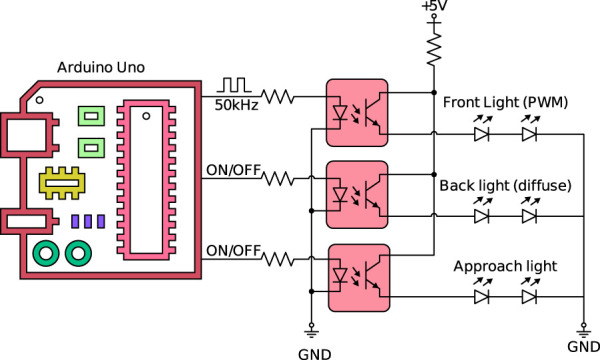
Microcontroller-based light system.

Figure 4 shows the illumination system connected to a microcontroller, which is commanded via USB with the main computer via custom commands from the Python script to switch on/off the individual lights or change their illumination levels (PWM percentage).

The approach to analyzing a hole tilt using front and back circle coordinates was developed and tested in previous work (Morsi et al., 2023) (also presented in Analysis stage), including a custom circle detection optimization algorithm to achieve sub-pixel accuracy for the estimation of the center and radius of the circle; it was applied for the measurement of the tilt of drills in a test block. To transfer this analysis to the current problem, the designed tool needs to accommodate light sources to allow the detection of the front and back of the mounting hole pairs (i.e., 1–1b, 2–2b,...). Therefore, a U-shaped tool design was created specifically to allow for the subsequent capture of the front and back holes of the slat support from the inspection camera using the front light and back light, respectively.

The motion control of the robot for centering objects in the image, or fixing the orientations, is implemented using a Proportional–integral–derivative (PID) approach working on visual feedback from the relevant camera. This allows the part to be stationed in any reachable place, avoiding the need for calibrated, fixed positions.

**FIGURE 5:**
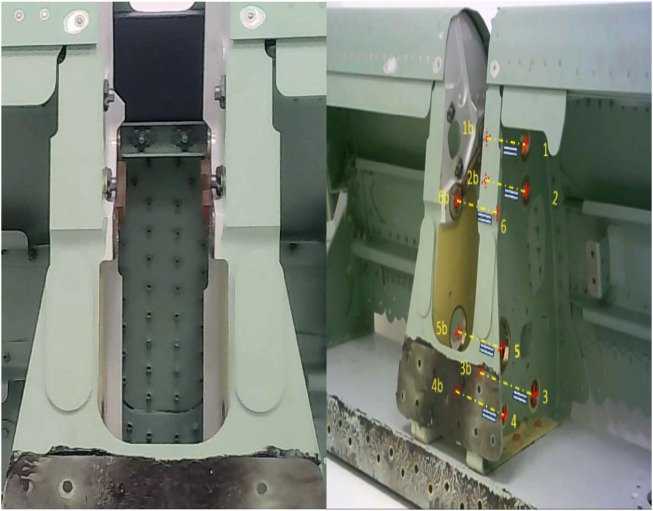
Shape to be detected (mounting slot for the slat actuator).

To initiate the inspection process, the defined parameters of the system are as follows:

• The template used in the system to approach the spar (a frontal view of the support to be found, i.e., Figure 5).
• Approximate the minimum and maximum radii for each hole (in pixels).
• Rough offset to prevent the front camera and back light from colliding with the spar (approximately 50 mm).

In our approach, we use ML techniques to solve the two steps needed in the image processing to locate the workpiece to be inspected (mounting slots for slat actuators along a wing spar):

• Locating the workpiece to be inspected in the image.
• Estimating the orientation of the camera with respect to the mounting slots.

A regular webcam mounted on the robot flange (with the camera axis roughly normal to the frontal surface of the flange) is used for this. This is called the *approach* camera as it is used to control the motion of the robot to approach the work piece before inserting the inspection tool into the wing spar structure. Figure 5 shows an example of the support slot to be found. The robot must center the approach camera (and consequently, the inspection tool) with respect to it and orient itself to be perpendicular to it.

#### 3.1.1 Positioning the robot tool with respect to the slot

For this particular application, Figure 5 was used as the template to be detected, representing a typical view of the target support slot. Both SIFT and U-SURF extractors were tested in combination with both brute-force and kNN matchers to identify the more effective system. The tests show U-SURF to be the best feature extractor (exploiting the fact that the target will not suffer relevant rotations), while kNN is the fastest matcher. Overall, the U-SURF extractor paired with the brute-force matcher demonstrated the highest detection accuracy.

This detector is used to calculate the pixel coordinates of the target in the image, which is then used to control the motion of the robot (using an empirically tuned PID approach) until the target is located at the center of the image and an adequate distance (determined by a correct apparent size in the image in pixels). After this centering approach, the orientation of the camera with respect to the target is now determined (and controlled) using a separate ML approach, which is described next.

#### 3.1.2 Estimating the orientation of the camera with respect to the target

The robot needs to detect the slat actuator mounting slots and orient itself to be normal to the slot surface before inserting the inspection tool. ML is also used for this task, in this case, by training a dedicated algorithm estimating the relative orientation of the approach camera with respect to the target (mounting slot). Once this system is trained, the ML algorithm will operate in the following way:

1. Capture an image with the approach camera.
2. Extract the features of the image and input them into the pre-trained ML system.
3. A classification output is presented, representing the relative orientation.
4. According to the output orientation class, the robot rotates the tool by a small amount, correcting the orientation and trying to make it “centered” (normal to the target).

These steps are repeated until the ML algorithm indicates a “centered” orientation in several successive images. Then, the inspection tool can be safely inserted into the spar structure. This inspection tool holds a second camera (the “inspection” camera) that takes control to inspect the mounting holes present on the target slot.

In general lines, an ML system is designed through a series of steps:

• Unless a suitable training dataset already exists (which is unlikely in new applications), a large number of training examples must be generated and manually associated with the desired outputs for each example.
• Training examples can be *augmented* to increase their number and/or to emulate possible variations occurring in real-world operations.
• Related literature is reviewed to identify the possible algorithmic approaches to be considered.
• Possible *features* that are more likely to let the ML algorithm determine how to produce the correct outputs from the inputs are reviewed (or designed), i.e., features that contain relevant information about the problem.
• The selected features are extracted from the training dataset.
• The system is trained and tested to tune its parameters so that it provides the expected output.

Following a literature survey of possible detection algorithms (Vailaya et al., 2002; Luo and Boutell, 2004; Luo and Boutell, 2005; Shima et al., 2017), random forest, Bayesian, and support vector machine (SVM) approaches were initially selected for their computation time and feasibility and the ability to work with relatively few training examples. The machine learning algorithms were tested on features extracted from images, such as Figure 5. The extracted features selected include Sobel masks, Histogram of Oriented Gradients (HoG), and Harris corners (Harris and Stephens, 1988; Chaple et al., 2015; Akshaya Devi and Arulanand, 2023).

**FIGURE 6:**
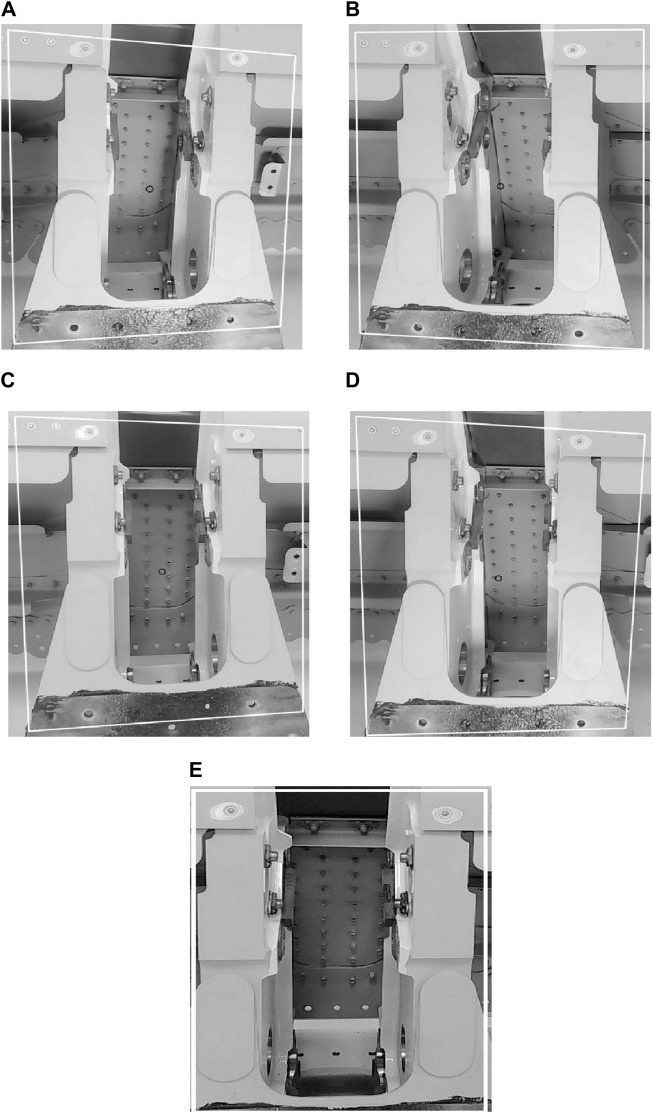
Example workpiece orientations for each considered class. **(A)**: Heavy left, **(B)**: Heavy right, **(C)**: Slight left, **(D)**: Slight right, **(E)**: Centred.

A training dataset has been created, with approximately 2,000 images exhibiting different orientations of the workpiece. Determining the orientation has been set up as a classification problem, where the considered orientations are shown in Figure 6. The robotic system should only insert the inspection tool into the wing spar structure once the orientation of the workpiece is correctly aligned, i.e., “centered,” as shown in Figure 6E, with the approach camera optical axis approximately normal to the workpiece.

To select a suitable machine learning system capable of estimating the camera orientation with respect to the slot surface, training using k-fold cross-validation is performed on SVM, random forest, and Bayesian algorithms. K-fold cross-validation is a technique that helps avoid overfitting (Peco Chacón et al., 2023). These algorithms are supplied with the same feature vectors, containing Harris corners, Sobel masks, and HoG values. The tests showed that the SVM algorithm outperformed the other approaches, achieving 94% accuracy. Finally, the previous detector approach and this trained SVM are integrated into the system’s control approach.

### 3.2 Control approach

**FIGURE 7:**
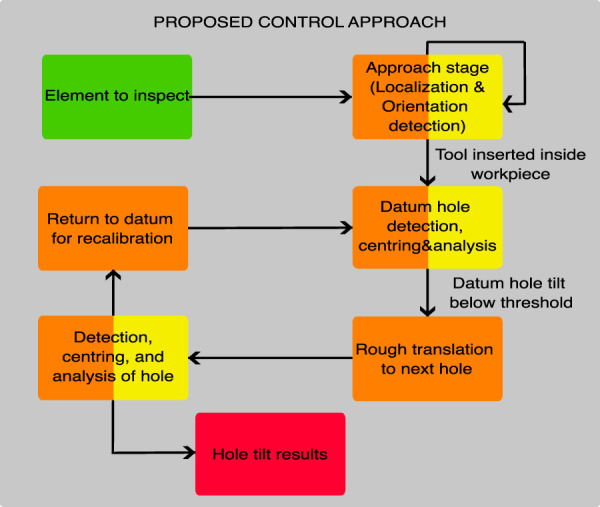
Proposed control framework.

RoboDK is used to run a Python script containing the control program, connected to the main robot’s controller via a standard Ethernet connection used to send motion commands. The control approach follows the design proposed by Morsi et al. (2023), loosely based on FSM. This design approach allows conceptual states to be programmed and easily modified, removed, or added to adapt to other similar inspection applications. Figure 7 shows the top-level flow diagram, where the orange blocks represent robotic motion steps while the yellow blocks represent image-processing steps (blocks with a combination of green and yellow represent a mix of image-processing steps and robotic motion steps). The execution is initialized at any start position close enough to the element to be inspected to make it appear in the approach camera image. Then, the system initiates an approach control to center the element to be inspected in the image.

#### 3.2.1 Approach stage

To commence the inspection process, the robot is initialized with the tool roughly parallel to the ground (no need for calibrated orientation). The system initiates the process by capturing an image with the approach webcam, followed by the use of the U-SURF matching subsystem (discussed in Section 3.1.1) to detect the template of the element to be inspected (slat actuator support). When the detection is successful, the system calculates the translation error between the template center in the captured image and the camera optical center (the center of the image). This error is then used in a proportional controller that calculates the robot’s motion appropriately to center the tool on the target element. Furthermore, the system calculates how close the tool is to the target by calculating the detected template bounding box area (inversely proportional to the distance between the camera and target) and comparing it to a pre-defined area to keep the tool at a safe distance from the target before attempting the insertion.

Once the target is centered in the approach camera and at the correct distance, the pre-trained ML orientation subsystem (discussed in Section 3.1.2) is used to correct the orientation until the approach camera is perpendicular to the target. As this re-orientation can take the target away from the center of the image, the centering and orientation steps are alternated. This process ends up with the approach camera (and therefore, the inspection tool) centered on the target, at the desired distance, and normal to it.

**FIGURE 8:**
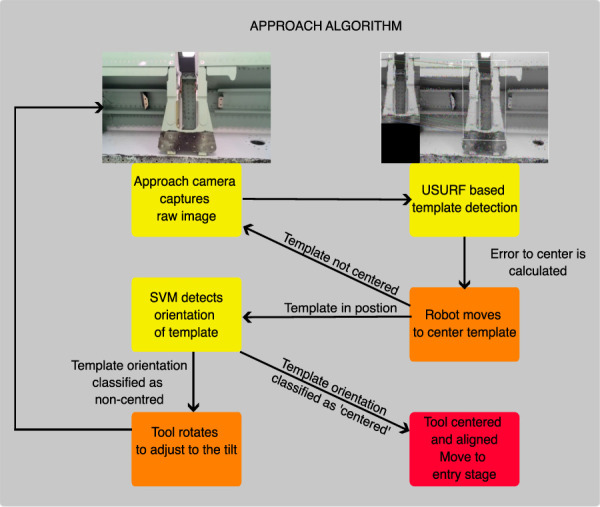
Approach algorithm.

Then, a pre-defined translation motion is made to insert the inspection camera (and backlight) into the spar; the C-shape of the tool leaves the inspection camera and frontal light on the right side of the support and the backlight on the left side, as shown in Figure 3. The control approach used to center the template in the approach camera image and correct the orientation is shown in Figure 8.

### 3.3 Entry stage

To ensure the safe insertion of the tool into the spar, the system makes a translation position in the flange Z coordinate axis (roughly along the approach camera optical axis) to enter the shape while attempting to detect the closest hole (number 5, which will be used as the reference, or *datum*, hole). The approach light is switched off, and the front inspection light is turned on to facilitate detection by the industrial camera. This aims to initialize the inspection process with the procedure starting within the vicinity of mounting hole 5 (Figure 2), which is the first hole expected to be detected by the industrial camera during the entry motion. The decision to select drill 5 as the datum is influenced by the relatively large diameter of the hole, which contributes to a lower misalignment error than in smaller holes, and its convenient location next to the entry point. Furthermore, this hole is positioned midway between other mounting holes, allowing a shorter trajectory to reach them to reduce accumulated error during motions. For each hole (including datum hole 5), very rough minimum and maximum radius values (in pixels) are used for faster detection while decreasing the chances of wrong detection.

**FIGURE 9:**
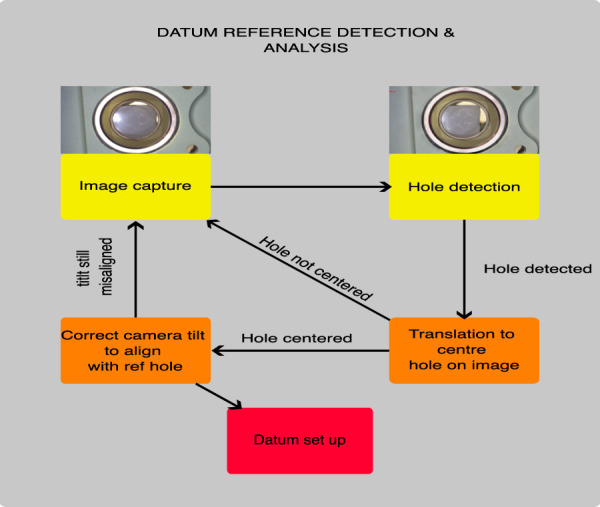
Datum reference detection and analysis.

The system keeps entering the wing spar until the hole is detected by the inspection camera for the first time; then, the insertion motion is terminated, and the hole-centering algorithm dictates the robot motion in the camera *x* and *y* directions to center the hole within the image so that the inspection camera optical axis is centered on the front hole center. This is controlled as before: the difference between the hole center and the camera center is the error input to a PID controller, with the output being the relative translation motion values of the robot. The PID controller ensures a smooth trajectory during centering and avoids overshooting (which could cause small collisions in those holes close to the spar surfaces). The hole centering is mostly a replica of the one used for the approach. Figure 9 shows the datum algorithm approach.

#### 3.3.1 Analysis stage

**FIGURE 10:**
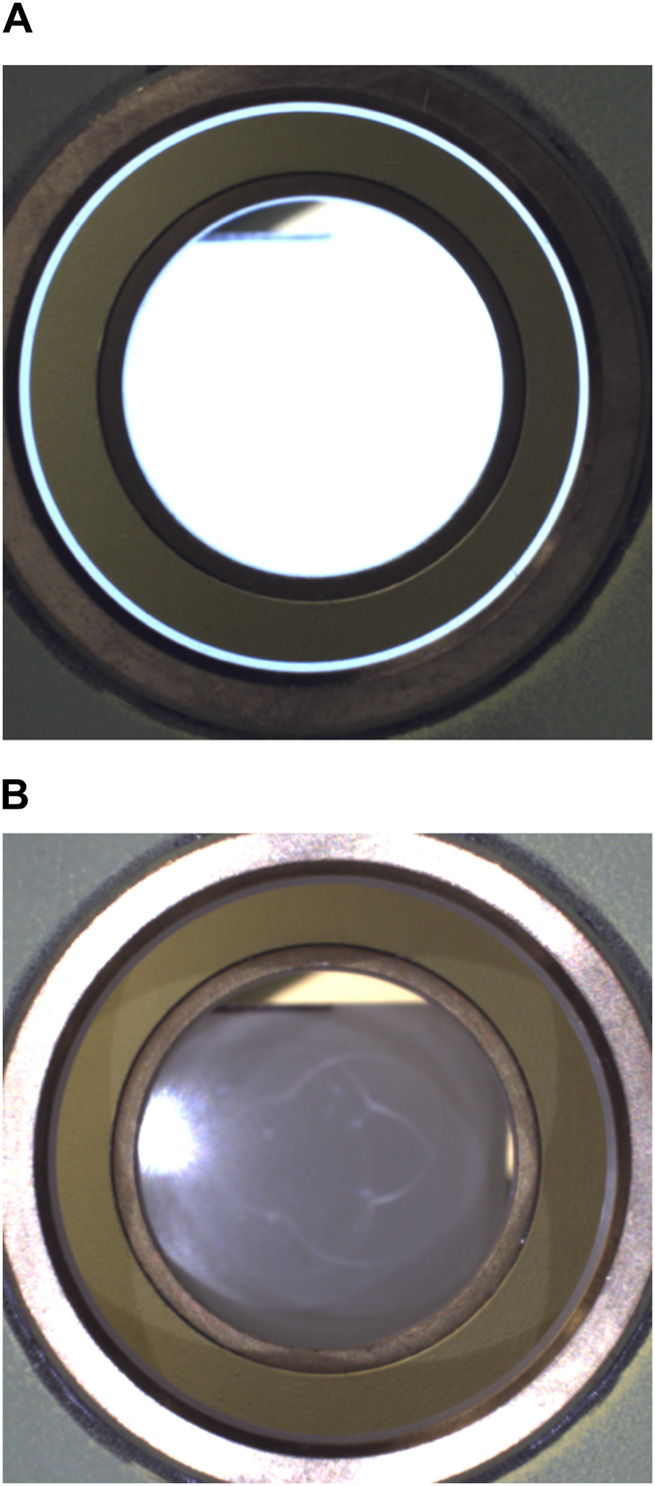
**(A)** Image used for back-hole detection (back light on). **(B)** Image used for front-hole detection (front light on).

After any front hole has been centered in the inspection camera image, the analysis module is used to compute the hole tilt with respect to the camera’s optical axis. The process commences with the back light on, allowing the camera to capture an image where the back hole is highlighted (such as that shown in Figure 10A), from which the center and radius values (*x*, *y*, *r*) of the back hole circle are extracted in pixels. Then, the back light is turned off, and the front light is switched on, producing a correct image of the front of the hole (such as that shown in Figure 10B). The search for the front hole is now restricted to an area surrounding the back hole to allow for faster and more robust detection of the front hole, obtaining its corresponding center and radius. After the detection of the front and back holes, a custom-optimized circle search algorithm is used to estimate the circle center and radius with a pixel error of 0.4 (Morsi et al., 2023). Using the front- and back-circle coordinates (x, y, and r), the tilt angle *α* is calculated using Eq. 1 derived from the pinhole model (discussed by Morsi et al. (2023)):

$$\alpha =\tan^{-1}\left(\frac{\left(y_{c}-y_{t}\right)}{f\cdot K}\frac{r_{t}}{\left(r_{t}-r_{c}\right)}\right),$$ (1)

where

• *r*_*t*_: front-circle radius (pixels).
• *r*_*c*_: back-circle radius (pixels).
• *y*_*t*_: front-circle coordinate (pixels).
• *y*_*c*_: back-circle coordinate (pixels).
• *f*: focal length (mm).
• *K*: image sensor physical resolution (pixels/mm).

**FIGURE 11:**
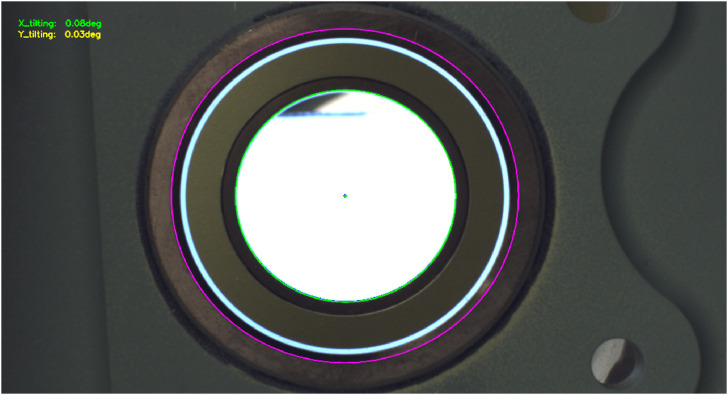
Example of drill-hole tilt analysis combining the front- and back-hole detection.

The datum hole is used as the tilt reference for all of the mounting holes. If the measured datum hole tilt is greater than 0.2° in either the x or y direction, the system proceeds to reorient the camera to align the optical axis with the datum hole axis until the observed tilt is less than 0.2° (as shown in Figure 11). As this may mis-center the frontal hole in the image, a centering step is also taken. This reorientation/recentering process is executed iteratively until the camera axis is aligned with the datum-hole axis. The selected threshold of 0.2° is enough to satisfy the requirements; the visual analysis can potentially do better than that (theoretically, down to 0.03° as discussed in Results), but the time it takes to make tiny corrections greatly increases, and eventually, the robotic arm precision limits get into play and are not able to reliably follow the relative motion control commands.

With the reference hole centered and aligned with the optical axis, the current robot pose is now memorized as the datum. If needed, 3D reconstruction can now be used to position the front and back holes with respect to the robot base, although this is not the target of this application.

Once the datum pose is set up, the system initiates the search and analysis of the remaining mounting holes. The image plane is now roughly normal to the surface containing the remaining holes, so the approach executes a rough translation (in the camera frame) to the vicinity of the next hole to be inspected. The precision needed to indicate the relative position of the next hole is within a few cm as it is enough that the next hole appears inside the inspection camera image; then, the controlled centering approach takes control to precisely center the frontal hole in the image. The rough relative position of the remaining mounting holes with respect to the reference hole is determined by just using a simple ruler, and the results are given in Table 3. It is the same for all mounting slots to be inspected as any variations in the actual location of the holes in each specific slot will be below a millimeter. Strictly speaking, the rough relative position of the remaining holes would not be necessary as the robot could follow an exploration approach to locate the remaining holes, but it would greatly increase the inspection time, and a motion limit envelope would be necessary to avoid the inspection camera colliding with the inside of the spar.

Once the next hole is centered in the inspection camera image, the tilt analysis is run, and the tilt results are recorded. At this point, 3D reconstruction could be used to determine the position of the front and back holes with respect to the robot base, if desired.

Then, the robot returns to the recorded datum pose and repeats the reorientation process to cancel any misalignment due to the limited motion precision of the robot. It then travels to the vicinity of the next hole and starts the centering and tilt analysis. This iterative approach is repeated until the tilt of all mounting holes is measured.

## 4 Results and discussion

The system is able to autonomously detect and analyze all the drilled holes on the support slot in real experiments, regardless of the relative location of the spar itself (note in Figure 3 that the spar is just resting on a pallet, obviously uncalibrated) as long as the slot is within the view of the approach camera (which covers approximately 2 m wide and 1.5 m tall at the work distances).

Table 1 presents a comparison between the measured tilt (in degrees) of the robot for each pair of left-right mounting holes in the support slot (determined with respect to the datum hole 5) and a meteorological measurement of the same tilt. This measurement is carried out manually using a clock gauge, which measures the tilt of a steel bar resting at the lower part of both holes in a pair. This is an indirect measurement performed by setting the height on one extreme of the bar with respect to a flat steel table as 0 and then measuring the height difference (in mm) with the other extreme of the bar with respect to the table surface. The tilt angle is then calculated as the arc-tangent of the height difference over the bar length. This procedure is accurate down to 0.08°. As the robot tilt measurements are relative to datum hole 5, the hand-measured tilt of hole 5 is subtracted from the tilt of other holes to provide equivalent values. This manual measurement checks for just the vertical tilt, matching the Y tilt in the camera frame, and verifies the accuracy of only one direction. Similar hand-made measurements of the tilt in the horizontal direction could not be obtained with the equipment available. The manual measurement results indicate that all mounting holes exhibit tilts well below the usual requirement of 0.5° in the aerospace industry (at least in the measured *y*-axis). The observed differences between the robot-measured tilts and manual-measured ones are within ± 0.08°.

**TABLE 1:** Comparison of robot-measured and actual vertical-axis tilt values for each hole, in degrees.

Drill number	Robot-measured *y*-axis tilt	Actual *y*-axis drill tilt	Measurement error
Drill 1	0.04	−0.039	0.079
Drill 2	−0.02	−0.103	0.083
Drill 3	0.1	−0.094	0.194
Drill 4	−0.01	−0.048	0.038
Drill 6	0.09	−0.026	0.12

A comparison is also made between the tilt in the *y*-axis for the datum hole (number 5) before moving to inspect a new hole and after returning to the memorized datum after inspecting that hole. The results are presented in Table 2, averaged over three measurement runs. This reveals that the robot’s motion is not precise enough to keep an exact tilt reference, justifying the need for re-alignment after each new inspection to minimize this motion-related error.

**TABLE 2:** Comparison of hole 5 (datum) tilt before and after inspecting each new hole, in degrees.

Drill number	Before new hole inspection	After returning from inspection	Tilt change
Drill 1	−0.16	−0.2	0.04
Drill 2	−0.22	−0.24	0.02
Drill 3	−0.15	−0.2	0.05
Drill 4	−0.2	−0.17	−0.03
Drill 6	−0.19	−0.24	0.05

Morsi et al. (2023) reported that the tilt error when inspecting drill holes with a front and back circle distance of 10 mm was 0.04° (mostly resulting from the sub-pixel error when determining the center and radii of the hole circles); in this application, with an increased distance of 155 mm between the front and back holes of each pair, the inspection accuracy gets as low as 0.03°. This is the only tilt calculation error contributed by the approach; therefore, the remaining error up to the observed 0.08° can be the motion-related error, attributed to the robot motion limitations failing to keep a perfectly constant orientation in the motion from the reference hole to each of the remaining holes.

Table 3 summarizes the rough linear distances along the camera *x* and *y*-axes (corresponding to flange *z* and *x*-axes) used to reach each of the holes from the datum hole (the precise adjustments to center each hole in the image are then carried out using visual feedback control on the detected holes, as discussed). The longest distances correspond to holes 3 and 6 (100 and 120 mm, respectively), which also happen to be the holes causing the biggest misalignment in the datum tilt when returning from their inspection. That reinforces the idea of using as the datum a hole that minimizes the maximum distance to travel to the remaining holes.

**TABLE 3:** Rough translation of the robot in the camera *x* and *y*-axes to reach other holes from the datum hole, in mm.

Drill number	Robot motion in the *z*-axis (mm)	Robot motion in the *x*-axis (mm)	Total distance moved (mm)
Drill 1	−70	−20	72.8
Drill 2	−50	−20	53.8
Drill 3	50	90	102.9
Drill 4	50	0	50
Drill 6	−120	0	120

## 5 Conclusion

In this paper, we present an initial proposal for a robotic system using visual feedback to measure the tilts of a group of related mounting holes relative to one of them selected as the datum. The system is based on the hole tilt inspection approach discussed by Morsi et al. (2023) and extended to an intricate structure like the mounting slot for a wing slat actuator. Other than designing a custom tool to hold the different lights and the approach and inspection cameras in a way that allows for their insertion within the wing spar structure, the approach required minimal modifications. The control strategies were developed in a simulated environment on RoboDK and run as Python scripts (which then interact with the robot controller), providing full flexibility.

Machine learning has been used to calculate the relative position and orientation of the target to be inspected (the support slot) to control the robot approach stage and could be extended to different targets for other inspection tasks.

All robotic motion is driven by visual feedback control, eliminating the reliance on calibrated positions. Therefore, the proposed system can be just mounted onto a cart and easily wheeled by a person to deploy it near the support to be inspected (it just needs to appear within the field of view of the approach camera, covering approximately 1 m at the operation distance); the use of a collaborative robot plus low operation speeds avoids the need for a protection cage. Ground-truth measurements demonstrate a system accuracy of 0.08° on this task. Robot motion limits are the main reason for preventing even better accuracy.

Further optimization to speed up the precise alignment motion required for inspecting the holes, i.e., centering the holes in the image and, especially, adjusting the datum tilt, would be beneficial. The current control PID approach converges slowly as it is adjusted to avoid overshooting (which might cause low-velocity collisions of the inspection camera with the inside of the spar). This can be accomplished by incorporating a better motion control model. Another aspect to improve is the machine learning techniques used for locating the inspection target during the approach stage; an adapted simultaneous localization and mapping (SLAM) approach (Temeltas and Kayak, 2008) deals with position and orientation in a more cohesive way.

## Data Availability

The original contributions presented in the study are included in the article/supplementary material, further inquiries can be directed to the corresponding author.
